# Novel self-expandable metallic stent with dumbbell-shape and spiral outer cover for malignant distal biliary obstruction

**DOI:** 10.1055/a-2420-7965

**Published:** 2024-10-08

**Authors:** Haruo Miwa, Yugo Ishino, Shotaro Tsunoda, Kazuki Endo, Ritsuko Oishi, Yuichi Suzuki, Shin Maeda

**Affiliations:** 1 26437 Gastroenterological Center, Yokohama City University Medical Center, Yokohama, Japan; 2 Department of Gastroenterology, Yokohama City University Graduate School of Medicine, Yokohama, Japan

Covered self-expandable metallic stent (SEMS) placement is effective for malignant distal biliary obstruction 1 ; however, it carries the risk of cholecystitis, pancreatitis, and stent migration 2 3 4 . The novel HILZO dumbbell-shaped, spiral cover stent (ABIS Inc., Hyogo, Japan) has a unique structure that prevents such complications ( Fig. 1 ). The dumbbell-shaped proximal end prevents stent migration. The belt-like gap in the spiral outer cover helps bile and pancreatic juice flow out, whereas the inner full cover prevents tumor growth. Herein, we report the first case of a malignant distal biliary obstruction treated using the novel HILZO stent ( Video 1 ).

**Fig. 1:**
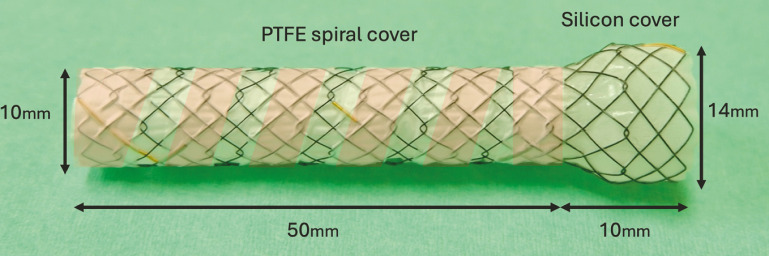
The novel HILZO dumbbell-shaped, spiral cover stent. The stent has a dumbbell-shaped proximal end, 14 mm in diameter and 10 mm in length, with a silicon cover. A full inner cover and spiral outer cover were attached to the stent body.

The novel HILZO stent with dumbbell shape and spiral cover was successfully deployed for the patient with distal malignant biliary obstruction.Video 1

An 88-year-old man with unresectable pancreatic cancer was referred to our hospital with obstructive jaundice. Initial biliary drainage was performed using a 7-Fr plastic stent; however, the patient developed cholangitis secondary to stent migration. After cholangitis improved with plastic stent exchange, SEMS placement was planned. As hyperamylasemia was observed, the novel HILZO stent was selected to prevent pancreatitis and stent migration. Cholangiography revealed a stricture, 2 cm in length, in the distal extrahepatic bile duct ( Fig. 2 ). An 8.5-Fr stent delivery system was smoothly advanced through the stricture and the stent was adjusted to position the 1-cm dumbbell-shaped proximal end on the hepatic side of the stricture. The stent was gradually deployed by an assistant carefully retracting the outer sheath. Resistance during stent release was not strong. A yellow marker was useful for detecting the distal end of the stent. Finally, the stent was successfully placed 1.5 cm above the biliary stricture to the duodenum. ( Fig. 3 ). The patient was discharged two days after SEMS placement without any complications.

**Fig. 2:**
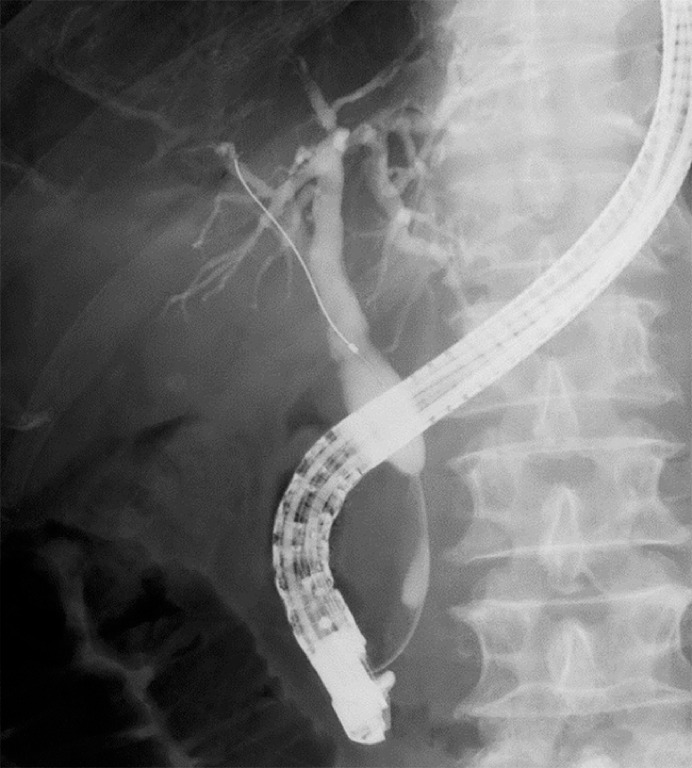
Cholangiography revealed a stricture, 2 cm in length, in the distal extrahepatic bile duct.

**Fig. 3:**
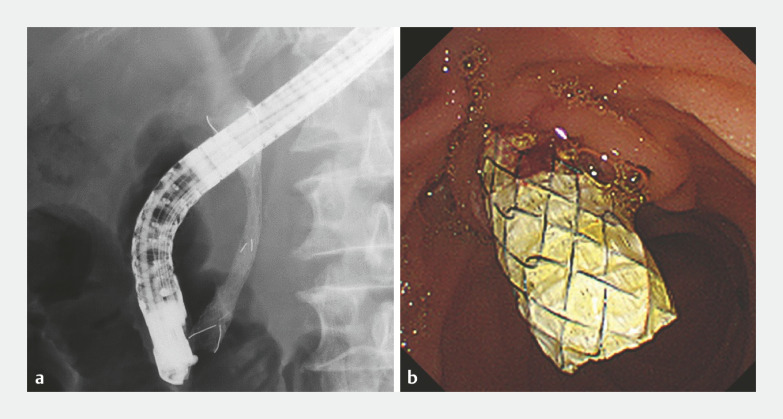
Fluoroscopic and endoscopic images after the novel HILZO stent placement. **a** The stent was successfully placed 1.5 cm above the biliary stricture to the duodenum. **b** The distal end of the stent was exposed from the papilla.

To the best of our knowledge, this is the first case in which the novel HILZO stent with dumbbell shape and spiral outer cover was placed. Theoretically the stent is designed to prevent migration and avoid pancreatic or cystic duct obstruction; however, this has not been proven. Prospective clinical trials are required to examine the effectiveness of this stent.

Endoscopy_UCTN_Code_TTT_1AR_2AZ

## Notes

Endoscopy E-Videos https://eref.thieme.de/e-videos
*E-Videos* is an open access online section of the journal *Endoscopy* , reporting on interesting cases and new techniques in gastroenterological endoscopy. All papers include a high-quality video and are published with a Creative Commons CC-BY license. Endoscopy E-Videos qualify for HINARI discounts and waivers and eligibility is automatically checked during the submission process. We grant 100% waivers to articles whose corresponding authors are based in Group A countries and 50% waivers to those who are based in Group B countries as classified by Research4Life (see: https://www.research4life.org/access/eligibility/ ). This section has its own submission website at https://mc.manuscriptcentral.com/e-videos .
